# Development of Screen-Printed Electrode Biosensor for Rapid Determination of Triglyceride Content in Coconut Milk

**DOI:** 10.1155/2020/1696201

**Published:** 2020-05-06

**Authors:** D. Manoj, Ishita Auddy, Shubham Nimbkar, S. Chittibabu, S. Shanmugasundaram

**Affiliations:** Indian Institute of Food Processing Technology, Thanjavur, 613005 Tamil, India

## Abstract

The screen-printed electrode biosensor was developed for triglyceride determination in coconut milk. The biosensor was developed by adding lipase, glycerol-3-phosphate (GPO), and glycerol kinase (GK), which is immobilized to a gelatin solution. The concentration of triglyceride is found to be linear to the current produced. The developed screen-printed electrode biosensor showed the optimum response for pH 7.0, 45 mg amount of gelatin, 2.5% glutaraldehyde concentration solution. The developed biosensor was able to find triolein concentrations 0.1 to 1.5 mM. The correlation obtained between these two methods was 93% which was found to be good.

## 1. Introduction

Food quality is the most important factor for any manufacturing requirement because food consumed undergoes contamination from the farm to the industrial manufacturing process. Chemical and microbiological analysis is being periodically done to check food quality, which requires sample preparation, pretreatment being required and most expensive process. Therefore, biosensors can resolve all this by checking quality quickly and at reception or a place where the quality needs to be detected [1]. The recent trend in analytical chemistry is going towards developing electrochemical devices, which are easy for portability, rapid detection, cost-effectiveness, selectivity, sensitivity, less space requirement, and minimal power [2]. Three-electrode systems have been developed to find the triglyceride content [3, 4]. The three-electrode system is difficult for portability and carrying to a measurement point because the three-electrode system contains three electrodes fabricated separately and needs 10 ml of sample to analyze the quantity of any compound present in the sample.

Screen printing technology is a well-established technique for fabrication of electrochemical sensors, which have high selectivity and sensitivity [5]. Screen-printed electrodes can be made from rigid substances to flexible materials as it can be printed on a flexible PET film as substances. Screen-printed electrodes not only reduce the cost of manufacturing but can also be made of a variety of electrode materials that can measure in a highly reproducible manner [6]. Electrochemical analysis, such as amperometric detection, has increasing popularity in detecting the quality of food products because of the very little time needed for sample preparation and detection of low amounts [7].

Coconut milk is an aqueous solution which is used in cuisines in the south and various parts of the world for various industries including bakeries, confectionaries, ice cream, and biscuit industries to enhance the taste and flavour of various products all around the world, which is extracted from solid endosperm of coconut [8, 9]. The coconut milk is highly susceptible to both chemical and biochemical spoilage that occurs through oxidation due to the presence of high oil content in it. The oxidation occurs through the breakdown of triglycerides into glycerol and free fatty acid, which is catalyzed by lipase enzyme. As there will be triglyceride breakdown, there is an increase in free fatty acid content in coconut milk. Triglycerides are natural fats made up of a total of three fatty acids and one glycerol molecule [4, 10]. Therefore, the amount of triglyceride content will be helpful to determine the quality of coconut milk, as triglycerides break down, then free fatty acid content will be increased [11]. In this research work, a screen-printed electrode biosensor was developed to detect the amount of triglyceride content present in coconut milk.

## 2. Materials and Methods

### 2.1. Reagents and Materials

All reagents were purchased from Sigma Aldrich-Merck (Bengaluru, India) of analytical grade: C. rugosa lipase (E.C.3.1.1.3), glycerol-3-phosphate oxidase (Aerococcus viridans) (E.C.1.1.3.21), glycerol kinase (Cellulomonas sp.) (E.C.2.7.1.30), triolein (Y0001113), Triton X-100 (9002-93-1), and ATP. Fresh coconut milk was extracted from fresh coconut meat [12]. The sodium phosphate buffer solution was prepared, as described [13].

Potentiostat/Galvanostat/impedance analyzer (PalmSens 4) was purchased from PalmSens (Netherlands). For the screen-printed electrode, the working electrode was made of carbon, the reference electrode is made of Ag/Ag Cl, and the counter electrode is made of carbon with no surface modification and the supporting material was alumina ceramic which was purchased from Class One Systems (New Delhi, India) and used for amperometric detection of triglycerides.

### 2.2. Methodology for the Development of the Screen-Printed Electrode Biosensor

**Figure 1:**
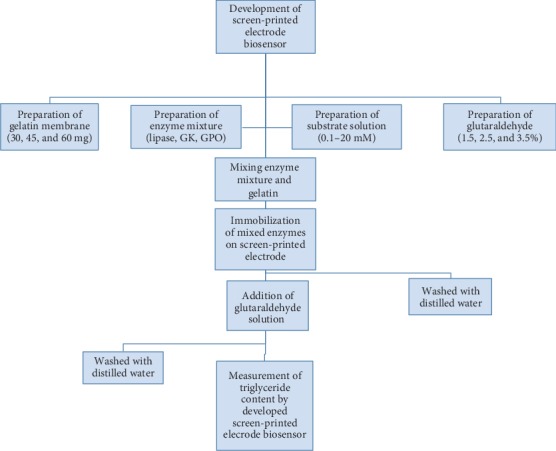
Process flowchart for the development of the screen-printed electrode biosensor.

The methodology for the preparation of screen-printed electrode is explained in Figure 1.

### 2.3. Preparation of Gelatin Membrane

Gelatin and BSA (30 mg) were added to 300 *μ*l of 0.1 M sodium phosphate buffer solution, and then, 31.5 *μ*l of MgCl_2_ was added to the mixture. The prepared solution was kept in the atmosphere for 10 min, and it was homogenized. A mixture of lipase, GPO, and GK has been coimmobilized into gelatin membrane solution through glutaraldehyde cross-linking. Here, one –CHO group was bound to –NH_2_ groups of BSA and another –CHO group of glutaraldehyde linked to –NH_2_ group of the enzyme [14].

### 2.4. Preparation of Triolein Solution

Triolein was used as a substrate for the enzyme lipase, GPO, and GK. The solution was prepared at nine different concentrations from initial concentration 0.1 mM to final concentration 20 Mm. The solution was prepared using a 0.1 M sodium phosphate buffer solution and stored at the refrigerated condition for further use [3].

### 2.5. Assay of a Mixture of Lipase, GPO, and GK

The enzyme solution of lipase, GK, and GPO was prepared by adding 1 mg of the enzyme into 1 ml of 0.1 M sodium phosphate buffer solution. From this, 300 *μ*l of lipase solution, 150 *μ*l of GK, and 60 *μ*l GPO were added in a ratio of 10 : 5 : 2 to form a mixed enzyme solution. The enzyme mixture also consists of ATP and MgCl_2_, which were prepared up to 10 *μ*l and added to the prepared enzyme mixture [4].

### 2.6. Construction of the Screen-Printed Electrode Biosensor

**Figure 2:**
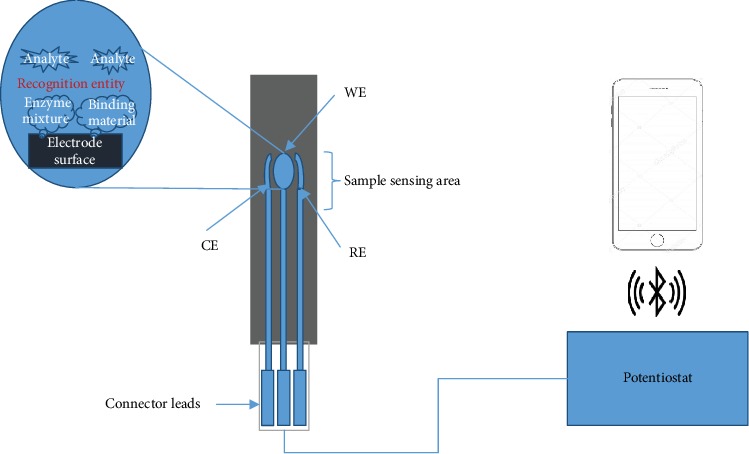
Schematic representation of the screen-printed electrode biosensor.

An amperometric biosensor for measuring triglyceride in coconut milk was constructed by adding 40 *μ*l of enzyme mixture, which contains lipase, GPO, and GK, mixed with 10 *μ*l of gelatin solution, and the enzyme mixture solution was then poured onto the working electrode and kept at 4°C for 30 min. The screen-printed electrode was then taken out, and again, 2.5% glutaraldehyde solution was poured onto the working electrode for 5 min for cross-linking, and then, the screen-printed electrode surface was washed using distilled water as explained in the flowchart in Figure 1. The constructed screen-printed electrode biosensor was taken for measurement to reaction cell connected to the potentiostat, as shown in Figure 2.

All the experiments were carried out at room temperature, and the current was measured for different triolein concentrations. The working electrodes were polarized at 0.4 V potential, and the current was measured amperometrically [15]. The reaction occurs as soon as triolein was added due to coimmobilized lipase enzyme, GPO, and GK. After triolein was added, it is then converted into dihydroxyacetone phosphate and H_2_O_2_. Then, H_2_O_2_ will split under potential applied through potentiostat, and then, the electrons were generated from H_2_O_2_ which passed to the counter electrode [16]. The reactions involved are as follows;

(1) $$\begin{array}{c} \text{triglyceride}\overset{\text{lipase}}{⟶}\text{glycerol}+\text{fatty acids}\\ \text{glycerol}+\text{ATP}\overset{\text{GK }{\text{MgCl}}_{2}}{⟶}\propto -\text{glycerol}‐3‐\text{phosphate}+\text{ADP}\\ \text{glycerol}‐3‐\text{phosphate}+{\text{O}}_{2}\overset{\text{GPO}}{⟶}\text{Dihydroxyacetone phosphate}+{\text{H}}_{2}{\text{O}}_{2}\\ {\text{H}}_{2}{\text{O}}_{2}⟶2{\text{H}}^{+}+{\text{O}}_{2}+2{\text{e}}^{-} \end{array}$$

### 2.7. Experimental Procedure

#### 2.7.1. Effect of the Amount of Gelatin on the Screen-Printed Electrode Biosensor

To study the effect of the amount of gelatin on the screen-printed electrode, three different gelatin concentrations (30, 45, and 30 mg) were taken, and enzyme mixture and glutaraldehyde concentration were kept constant. The working electrode was immobilized with different amounts of gelatin and response was noted.

#### 2.7.2. Effect of Glutaraldehyde Concentration on the Screen-Printed Electrode Biosensor

The three different varying concentrations of 1.5, 2.5, and 3.5% of glutaraldehyde solution were taken, and the effect on the screen-printed electrode biosensor response was found. The enzyme mixture and amount of gelatin concentration were kept constant.

#### 2.7.3. Effect of pH on the Screen-Printed Electrode Biosensor

Reaction buffer solution with pH ranges from 4.0 to 9.0 was made to study the effect on screen-printed biosensor response. The citric acid solution was added to 10 ml of sodium phosphate buffer solution to maintain the low pH range from 4.0 to7.0, and for high pH from 7.0 to 9.0, glycine solution was added. Enzyme mixture, amount of gelatin, and concentration of glutaraldehyde solution were kept constant. The output current for each pH condition was noted for a 5 mM triolein solution.

#### 2.7.4. Development of the Correlation between Triolein and the Output Current

For the readings for output current produced during the electrochemical reaction between enzymes, the sample was taken using a biosensor, and the amount of triglyceride was calculated using an empirical relation obtained.

#### 2.7.5. Triglyceride Measurement in Coconut Milk

To find the triglyceride content in coconut milk, 5 ml of milk was mixed with 10 ml of 0.1 M sodium phosphate buffer. Triton X-100 solution was added into this mixture, as it acts as an emulsifying agent.

#### 2.7.6. Validation of the Developed Screen-Printed Electrode Biosensor

Validation of the developed biosensor was done by using ultrahigh-performance liquid chromatography (UHPLC). UHPLC system (Shimadzu Corporation, Japan) equipped with column: Shim-pack XR-ODS III (100 × 2 mm, 2.2 *μ*m particle size), column temperature 40°C, flow rate 0.3 ml/min, injection volume 5 *μ*l was used. Mobile Phase ACN/MeOH/THF (40 : 40: 20 *v*/*v*/*v*) with the injection volume was 40 *μ*l, and samples were eluted at a flow rate of 1 ml/min.

## 3. Results and Discussion

### 3.1. Effect of the Amount of Gelatin on Biosensor Response

**Figure 3:**
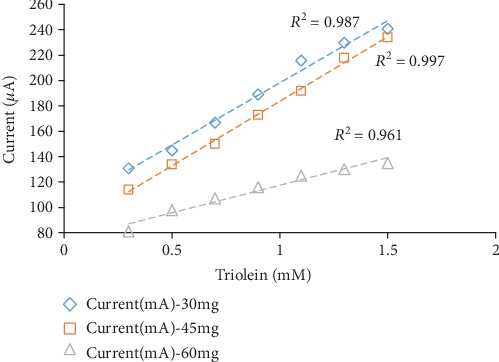
The effect of the amount of gelatin concentration on biosensor response.

Among the three different amounts of gelatin, 45 mg was found to be giving the best results for biosensor response, linearity, and *R*^2^ values. The results obtained are given in Figure 3. In 30 mg gelatin concentration, the substrate may leach out easily, and a higher concentration of 60 mg gelatin bioactive layer increases and makes it harder for the substrate to pass through a gelatin membrane. Thus, lower *R*^2^ and biosensor responses were obtained. A previous research also found a similar kind of result for the gelatin membrane [14], polyvinyl chloride membrane [17], and cellulose acetate membrane [18].

### 3.2. Effect of Glutaraldehyde Content on Biosensor Response

**Figure 4:**
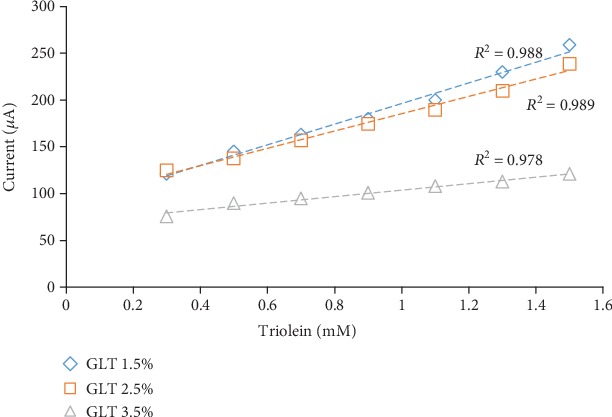
The effect of glutaraldehyde content on biosensor response.

The optimum results were obtained for 2.5% glutaraldehyde concentration, which is shown in Figure 4. In the case of low glutaraldehyde concentration of 1.5% which made a substrate to leak out easily from the gelatin membrane due to fewer cross-linking with gelatin membrane and for a high concentration of 3.5%, biosensor response was less due to more cross-link, which prevents the substrate from passing through a gelatin membrane. Similar results were obtained for acrylamide determination for French fries [19] and serum triglyceride determination [3].

### 3.3. Effect of pH on Biosensor Response

**Figure 5:**
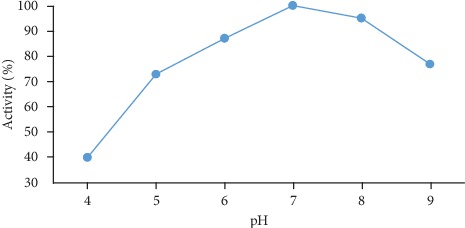
The effect of pH on biosensor response.

The buffer solution of different pH was made to see the effect on biosensor response, and it is shown in Figure 5. The biosensor response was found to be high for pH 7 and found to be low for other pH solutions. For pH 7.0, we can see the maximum current obtained which is plotted in the graph (current vs. pH). As a slight increase or decrease in pH could attribute to the changed micro environmental and differential folding of enzymes in the reaction, hence, the maximum current obtained has been accepted to have maximum activity. Different research has similar research for potentiometric biosensor 7.0 pH [20] and oxygen meter-based biosensor 8.0 pH [21].

### 3.4. Effect of Substrate Concentration and Development of Correlation between Triolein and Output Current

**Figure 6:**
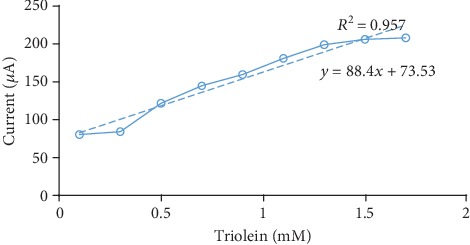
The calibration curve for the developed biosensor.

Different triolein concentrations ranging from 0.1 mM to 1.7 mM were taken to find a biosensor response. The experiment was conducted in 10 ml final reaction volume in 0.1 M phosphate buffer, in which pH is 7.0. The graph was plotted for a prepared biosensor. The linearity was obtained from 0.1 mM to 1.5 mM defined by the equation *y* = 88.417*x* + 73.536 (*R* = 0.952), which is shown in Figure 6. There is a deviation from linearity for higher concentrations which may be due to an insufficient amount of dissolved oxygen, which is cosubstrate to an enzyme or a limited amount of enzymes in bioactive material. Similar results were obtained for PVC membrane-based biosensor (5-2.1 mM) [17], silicon-based biosensor (0.2-2.1 mM), cellulose acetate-bound enzyme-based biosensor (0.2-3.5 mM) [20], and dissolved oxygen biosensor (5-2.0 Mm) [22].

### 3.5. Reproducibility

The reproducibility was tested for a total of three average standard solutions, which contain the same amount of triolein concentration of 0.5 mM. The standard deviation, variation of the coefficient, and average value were calculated as 0.083 × 10^−3^ mM, 11.97% (*n* = 3).

### 3.6. Validation of the Developed Biosensor

**Figure 7:**
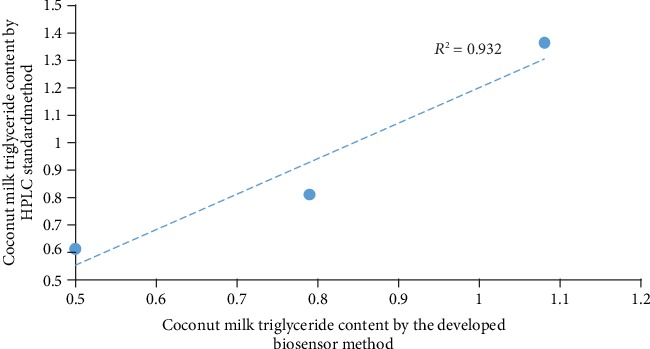
Correlation between the developed biosensor method and the standard HPLC method.

To study the accuracy of the present method, triglyceride detection from the high-performance liquid chromatography method [23] for coconut milk with different storage periods along with the present method was performed. The values obtained by both methods showed a good correlation *r* = 0.93, which is shown in Figure 7.

### 3.7. Detection of Triglyceride Content in Coconut Milk

The developed screen-printed electrode biosensor was applied to detect the amount of triglyceride in coconut milk. 5 ml of milk was mixed with 10 ml of 0.1 M sodium phosphate buffer. Then, the solution was taken into a reaction mixture, and the output current was measured for the 5^th^, 10^th^, and 15^th^ hour. The triglyceride content of the same coconut sample was measured using the UHPLC method. The results obtained by both methods were compared in Figure 7.

## 4. Conclusions

An amperometric screen-printed electrode biosensor was constructed to find the triglyceride content in coconut milk. The enzyme lipase, GK, and GPO were immobilized into a gelatin membrane. Then, the enzyme mixture was poured onto the working electrode. Then, the screen-printed electrode was connected to potentiostat for measurement of output current. The current measured was linear to the amount of triolein concentration.

## Data Availability

The data used to support the findings of this study are included in the article.
